# Population genetics of forest type of *Trypanosoma congolense* circulating in *Glossina palpalis palpalis* of Fontem in the South-West region of Cameroon

**DOI:** 10.1186/1756-3305-7-385

**Published:** 2014-08-20

**Authors:** Gustave Simo, Pythagore Soubgwi Fogue, Tresor Tito Tanekou Melachio, Flobert Njiokou, Jules Roger Kuiate, Tazoacha Asonganyi

**Affiliations:** Molecular Parasitology and Entomology Unit, Department of Biochemistry, Faculty of Science, University of Dschang, Cameroon, PO Box 67, Dschang Cameroon; Department of Animal Biology and Physiology, Parasitology and Ecology Laboratory, Faculty of Science, University of Yaoundé 1, P.O. Box 812, Yaoundé, Cameroon; Department of Biochemistry, Faculty of Science, University of Dschang, P.O. Box 67, Dschang, Cameroon; Faculty of Medicine and Biomedical Science, University of Yaoundé 1, P.O. Box 1364, Yaoundé, Cameroon

**Keywords:** Animal African Trypanosomiasis, *Trypanosoma congolense* forest, *Trypanosoma congolense* savannah, Microsatellite, Population genetics

## Abstract

**Background:**

Genetic variation of microsatellite loci is a widely used method for the analysis of population genetic structure of several organisms. To improve our knowledge on the population genetics of trypanosomes, *Trypanosoma congolense* forest and savannah types were identified in the mid-guts of *Glossina palpalis palpalis* caught in five villages of Fontem in the South-West region of Cameroon. From the positive samples of *Trypanosoma congolense* forest, the genetic diversity and the population genetic structure of these parasites were evaluated.

**Method:**

For this study, pyramidal traps were set up during three entomological surveys and 3347 tsetse flies were collected, dissected and 1903 midguts collected. DNA was extracted from midguts and specific primers were used to identify *Trypanosoma congolense* forest and savannah. All *Trypanosoma congolense* forest positive samples were characterized with seven microsatellite markers.

**Results:**

Microscopic examination revealed 25 (1.31%) mid-gut infections with trypanosomes while the PCR method identified 120 (6.3%) infections due to *Trypanosoma congolense:* 94 (78.33%) *Trypanosoma congolense* forest and 28 (21.77%) *Trypanosoma congolense* savannah. The trypanosome infection rates varied significantly between villages and years of capture. Menji recorded the highest infection rate (15.11%); and samples captured in 2009 were more infected (14.33%). The microsatellite markers revealed a genetic variability between *Trypanosoma congolense* forest populations of Fontem villages and 6.38% of mixed infections due to different genotypes of *T. congolense* “forest type”.

**Conclusion:**

Our data on the population genetics play in favor of a clonal reproduction of this parasite. The microsatellite markers used here showed a low genetic differentiation and an absence of sub-structuration (*F*_ST_ ≤ 0.0003) between *Trypanosoma congolense* forest populations of Fontem villages. However, the high *F*_ST_ value (*F*_ST_ ≥ 0.3911) between samples of the Democratic Republic of Congo and those of Fontem villages indicates low migration rates between trypanosomes of these subpopulations.

**Electronic supplementary material:**

The online version of this article (doi:10.1186/1756-3305-7-385) contains supplementary material, which is available to authorized users.

## Background

Trypanosomiasis is a disease caused by a parasite of the genus *Trypanosoma*. In Africa, many pathogenic trypanosomes such as *Trypanosoma congolense* (*T. congolense*), *Trypanosoma vivax* (*T. vivax*), *Trypanosoma simiae* (*T. simiae*) and *Trypanosoma brucei brucei* (*T. brucei brucei*) affect livestock. Most of these parasites are mainly transmitted by tsetse flies of the genus *Glossina*[1]. In sub-Saharan Africa, thirty-seven countries are affected by the Animal African Trypanosomiasis (AAT) or nagana, covering an area of about 9 million km^2^. This situation could partly be responsible for the malnutrition in Africa [2] because it is estimated that about 50 million cattle are exposed to the disease and US$35 million doses of trypanocides are used per year to overcome this pathology [3]. Controlling AAT would thus permit an increase in benefit for the agricultural and breeding industry to US$4.5 billion per year [4].

*T. congolense* is considered as the most pathogenic trypanosome species of animals [5]. During the last decades, several studies have been carried out in order to understand the epidemiology of the diseases caused by *T. congolense* as well as the pathological manifestations resulting from infections caused by this pathogen [6, 7]. From these studies, important data were generated on the prevalence and the distribution of *T. congolense* in many parts of Africa [8, 9]. Despite these data, important knowledge on the biology, the genetic diversity and the population genetics of *T. congolense* remain to be elucidated. For instance, the reasons explaining the diversity of outcomes that are observed during *T. congolense* infections are not well understood although hypotheses on the genetic variability of trypanosomes have been proposed. Compared to other trypanosomes like for instance *T. brucei* subspecies where several studies have been undertaken on their genetic characterization in order to understand the epidemiological importance of these parasites [10–12], little investigation has been undertaken on the genetic characterization of different types of *T. congolense*. Previous studies characterizing *T. congolense* by isoenzyme electrophoresis reported a clonal reproduction in these parasites [13]. Recently, Morrison *et al.*[14] used microsatellite markers and revealed a mating system within *T. congolense* savannah type circulating in animals of West Africa region. With the same microsatellite markers, Simo *et al.*[15] generated data that suggested clonal reproduction within the forest type of *T. congolense* circulating in domestic animals of the South-West region of Cameroon. Most studies that investigated the genetic variability of *T. congolense* populations were undertaken on trypanosome strains isolated or circulating in vertebrate hosts such as cattle, goat, sheep, dog, and pig [14, 16]. Up till now, no investigation has been undertaken on the genetic characterization of *T. congolense* circulating in tsetse flies of different areas of Africa. For *T. brucei* infections for instance, several studies reported genetic differentiation between *T. brucei* that infects mid-guts and salivary glands of different species of tsetse flies of west, central and east Africa [17, 18]. Investigating the genetic variability of *T. congolense* circulating in tsetse flies may enable us to improve our knowledge on the population genetics of these parasites, and improve our understanding of the transmission of *T. congolense* between villages, and between tsetse flies and different vertebrate hosts.

In this study, we undertook the identification of *Trypanosoma congolense* in the mid-guts of *Glossina palpalis palpalis* of Fontem in the South-West Region of Cameroon. Thereafter, microsatellite markers were used to characterize the forest type of *T. congolense* in order to improve our knowledge of the population genetics as well as the circulation of these parasites.

## Methods

### Study area

**Figure 1:**
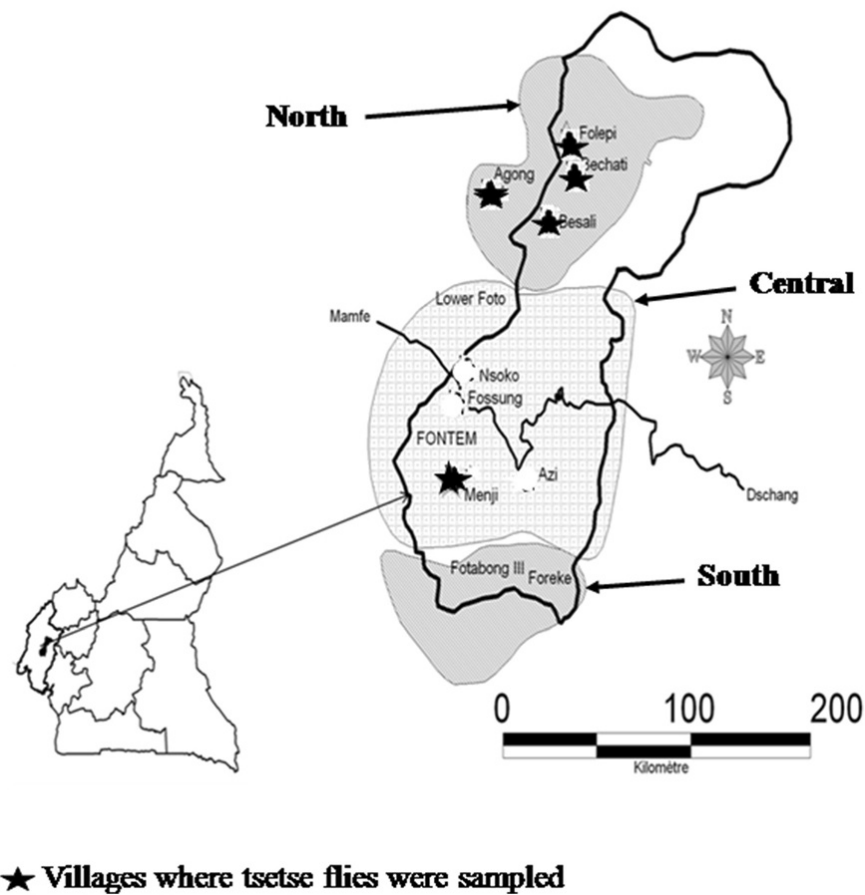
**Map showing the villages of the Fontem where tsetse flies were sampled.**

Fontem (5°40′12”N, 9°55′33”E) is located in the Lebialem division of the South-West Region of Cameroon. In this forested region, the climate is of tropical humid type and the topography is made up of hills and valleys through which several high speed rivers flow. The main activities of the Fontem population are agriculture, palm oil extraction, animal husbandry and poultry farming at a small scale. The present study was carried out in 5 villages; Besali, Bechati, Folepi, Agong and Menji (Figure 1).

### Sampling of tsetse flies

Tsetse flies were sampled during three entomological surveys that were conducted in November 2006 (end of the rainy season), April 2007 and April 2009 (end of the dry season). During each survey, pyramidal traps [19] were set in tsetse fly favorable biotopes (cocoa plantations, food crop farms, palm farms, oil extraction points, water points, roads and footpaths, homesteads, and pig sties). A total of 363 traps of which 140 in November 2006, 111 in April 2007 and 112 in April 2009 were set. For each of these traps, geographical coordinates were recorded with a Global Positioning System (GPS *etrex*, high sensitivity). Traps were visited twice a day during 4 days of capture in each village. Living tsetse flies were dissected in a drop of 0.9% saline solution using a stereo microscope and their mid-guts were examined under a light microscope (magnification × 100) for the presence of trypanosomes. Between dissections, the forceps were sterilized by immersing them in a solution of 0.1 molar sodium hydroxide for one minute and then in distilled water for another minute. After the microscopic examination, each mid-gut was collected and put in a microtube containing alcohol. In the field, the microtubes were conserved at ambient temperature; and at -20°C in the laboratory until DNA extraction.

### DNA extraction

DNA was extracted from tsetse mid-gut as described by Simo *et al.*[20]. In the laboratory, alcohol was evaporated during 60 min in an 80°C oven. Subsequently, 300 μl of Chelex 5% were added to each tube and the mixture vortexed for 10 min. Thereafter, two rounds of incubation took place; the first at 56°C for 30 min, and the second at 98°C for 60 min. The tubes were then centrifuged at 14000 rpm for 5 min and the supernatant (DNA extract) was collected and stored at -20 C or used directly for PCR.

### Identification of *Trypanosoma congolense*“savannah” and “forest” types

These trypanosomes were identified with specific primers: TCF1 (5′- GGACACGCCAGAAGTACTT-3′) and TCF2 (5′- GTTCTCGCACCAAATCCAAC-3′) for *T. congolense* forest [21]; and TCS1 (5′-TCGAGCGAGAACGGGCACTTTGCGA-3′) and TCS2 (5′-ACAATTAGGGACAAACAAATCCCGC-3′) for *T. congolense* savannah [22]. The amplification reactions were carried out as described by Herder *et al.*[23]. Each amplification reaction was performed in a total volume of 15 μl containing 20 pmol of each primer, 10 mM of Tris-Cl (pH 8.3), 50 mM of KCl, 1.5 mM of MgCl2, 1 mM of each dNTPs, 3 μl of DNA template and 1unit of Taq DNA polymerase. Amplification involved a pre-denaturation step at 94°C for 5 minutes followed by 40 amplification cycles made up of a denaturation step at 94°C for 30 seconds, a hybridization step at 60°C for 30 seconds, and an elongation step at 72°C for 1 minute. These were followed by a final elongation step at 72°C for 10 min. The amplification products were resolved by electrophoresis at 100 volts for 30 min on 2% agarose gel containing ethidium bromide. DNA bands were visualized under ultraviolet light.

### Genetic characterization of samples positive for the forest type of *T. congolense*

For this genetic characterization, all samples with a DNA sequence specific for the forest type of *T. congolense* were selected. For this characterization, the seven microsatellite DNA markers described by Morrison *et al.*[14] were used.

### Amplification of microsatellite DNA sequences

For this study, each PCR was carried out in a final volume of 25 μl containing 5 μl of DNA extract and 1.5 mM of MgCl_2_, 20 pmol of each primer, 1 mM of each dNTP and 1 unit of Taq DNA polymerase. For each sample, two PCR rounds were performed for each marker as described by Morrison *et al.*[14]. During these PCR rounds, two different pairs of primers were used. The first PCR round was carried out with two primers as described by Morrison *et al.*[14]. The amplification cycles contain one denaturing step at 95°C for 5 minutes followed by 30 amplification cycles. Each cycle had a denaturation step at 95°C for 50 seconds, an annealing step at 52°C for 50 seconds, and an extension step at 72°C for 1 minute. This was followed by a final extension at 72°C for 10 minutes. For the second PCR round (nested PCR), a 1/10 dilution of amplified products of the first PCR round was used as template, and the set of primers used were designed from a region located between the primers used for the first PCR round. The second PCR was performed in the same conditions as the previous.

After the two PCR rounds, 7 μl of each PCR product were checked by electrophoresis on 2% agarose gels for 30 min at 100 volts. The allele bands were then resolved and their sizes determined on 10% non-denaturing acrylamide gels. For agarose and acrylamide gels, bands were stained with ethidium bromide and visualized under UV light. After resolution of the amplified products on the polyacrylamide gel, a sample was defined as a multiple infection if it contained more than two alleles.

In addition to *T. congolense* forest positive samples of tsetse flies of Fontem, nine samples from *Glossina fuscipes quanzensis* of the Democratic Republic of Congo’s were analyzed as above and subsequently included as out groups during the population genetics study.

### Genetic data analysis

Population structure was assessed through Wright’s F-statistics [24]. *F*_IS_ measures the deviation from random union of gametes within subsamples and *F*_ST_ the deviation from random distribution of individuals between subsamples (and thus a measure of population differentiation). For the population structure analysis, all the individuals that displayed a single infection (78 individuals) were divided into four subpopulations according to their village of origin. Allelic richness was estimated with Fstat 2.9.4 software and tested through 10000 permutations within subsamples. Wright’s *F*-statistics were estimated using Weir and Cockerham’s unbiased estimators [25] in Fstat 2.9.4 software ([26] updated from Goudet [27]). The estimator used for *F*_IS_ was *f* and its significance was tested through 10000 permutations of alleles within subsamples. For *F*_ST,_ the estimator was θ and its significance was tested through 10000 permutations of individuals between subsamples. To get a general idea of individual distribution across the villages, an unrooted NJTREE was computed with MEGA 3.1 software [28] using the Cavalli-Sforza and Edwards [29] chord distances matrix, which were computed in the MSA software [30] and formatted in the software PHYLIP 3.69 [31].

### Statistical analyses

The Pearson chi-square test was used to compare the trypanosome infection rates when 80% of theoretical values were higher than 5. For the microscopic examination, the Pearson chi-square test with Yates correction was used to compare the trypanosome infection rates between years of capture because less than 80% of theoretical values were higher or equal to 3 and lower than 5. When 80% of theoretical values were lower than 3, Fisher’s exact test was used. These statistical analyses were performed using XLSTAT-PRO 3 software version 2009. The significant threshold was 5%.

## Results

### Entomological surveys

During the three entomological surveys, 363 pyramidal traps captured 3347 tsetse flies belonging to *G. p. palpalis* subspecies. Of these flies, 1903 (56.85%) of them that were still alive were randomly selected and dissected: 355 (72.15%) of 492 in 2006, 1241(56.33%) of 2203 in 2007 and 307 (47.08%) of 652 in 2009. Of the 1903 dissected tsetse flies, microscopic examination revealed 25 (1.31%) mid-gut infections with trypanosomes. The results of the microscopic examination according to villages and years of capture are reported in Table 1. No significant difference was observed between microscopic examination results (*P = 0.19318* between villages; *P = 0.246* between years of capture).

**Table 1:** **Infection rates of** ***Trypanosoma congolense*** **in the mid-gut of** ***Glossina palpalis palpalis*** **of different villages of Fontem**

Localities	NC	NE	T + (%)	Number of positive PCR (%)		
				TCF	TCS	NP
Agong	25	22	0 (0)	1 (4.45)	0 (0)	1 (4.45)
Besali	40	9	0 (0)	0 (0)	0 (0)	0 (0)
Bechati	683	446	10 (2.24)	19 (4.26)	4 (0.89)	23 (5.15)
Folepi	2091	1148	10 (0.87)	45 (3.92)	8 (0.69)	52 (4.52)**
Menji	508	278	5 (1.8)	29 (10.43)	16 (5.75)	44 (15.82)**
Total	3347	1903	25 (1.31)	94 (4.93)	28 (1.47)	120 (6.3)
*P*			0.19318*	<0.0013	<0.00001	<0.0001

NC: Number of tsetse captured; NE: number of tsetse examined; TCF: *Trypanosoma congolense* forest; TCS: *Trypanosoma congolense* savannah; NP: number of tsetse flies with *T. congolense* (forest and/or savannah type) in their mid-guts; *P*: p-value; *P value not significant; **refer to villages where mixed infections were identified.

### Molecular identification of the forest and savannah type of *T. congolense*

The PCR targeting a multi-copy repeat sequence specific to trypanosome species revealed 120 (6.31%) *Trypanosoma congolense* infections: 28 (1.47%) were *T. congolense* savannah and 94 (4.93%) *T. congolense* forest. Menji recorded the highest infection rate of 15.82% (44/278), followed by 5.15% (23/446) for Bechati and 4.52% (52/1148) for Folepi (Table 1). No *T. congolense* savannah was identified at Agong. Comparing the infection rates between villages, there was significant difference for the forest type of *T. congolense* (*P < 0.0013*) and the savannah type (*P < 0.00001*) (Table 1). A significant difference was also observed (*X*^2^ = 41.389; *P < 0.0001*) when the infection rates of *T. congolense* (forest and savannah types) were compared between years of capture (Table 2). The highest infection rate was 14.33% (44/307) in 2009, followed by 5.16% (64/1241) in 2007 and 3.38% (12/355) in 2006. For the forest type of *T. congolense*, about 13.03% of dissected tsetse flies were infected in 2009 while 3.38% were infected in 2006/2007. There was a significant difference in the infection rates of *T. congolense* forest (*X*^2^ = 51.018; *P < 0.0001*) between capture years. For *T. congolense* savannah, no infection was observed in tsetse flies caught in 2006. However, about 1.77% and 1.95% of tsetse flies caught in 2007 and 2009 respectively had mid-gut infections with *T. congolense* savannah; a significant difference (*X*^2^ = 6.573; *P = 0.037*) was found for *T. congolense* savannah infection rates between these years of capture.

**Table 2:** **Infection rates of** ***Trypanosoma congolense*** **according to years capture**

Years of capture	NC	NE	T + (%)	Number of positive PCR (%)		
				TCF	TCS	NP
2006	492	355	1 (0.28)	12 (3.38)	0 (0)	12 (3.38)
2007	2203	1241	18 (1.45)	42 (3.38)	22 (1.77)	64 (5.16)
2009	652	307	6 (1.95)	40 (13.03)	6 (1.95)	44 (14.33)**
Total	3347	1903	25 (1.31)	94 (4,93)	28 (1.47)	120 (6.36)
*X* ^*2*^			2.804	51.018	6.573	41.389
*P*			0.246*	<0.0001	0.037	<0.0001

NC: Number of tsetse captured; NE: number of tsetse examined; T + number of tsetse flies found infected by the microscopy; TCF: *Trypanosoma congolense*“forest type”; TCS: *Trypanosoma congolense*“savannah type”; NP: number of tsetse flies with *T. congolense* (forest and/or savannah type) in their midguts; *X* ^*2*^: Chi-square; *P*: p-value; *P value not significant; **refer to the year where mixed infections were identified.

Two mixed infections of *T. congolense* forest and *T. congolense* savannah were found in two tsetse flies caught at Menji and Folepi in 2009.

### Genetic characterization of *T. congolense*forest type

This characterization was performed only on the 94 *T. congolense* forest positive samples. Of the seven microsatellites markers used in this study, no amplification was obtained for marker TCM3 while only 6.38% of these samples were amplified by TCM5 marker. These two markers were not considered for subsequent analyses. Out of the nine *T. congolense* forest samples from DRC, TCM5 amplified all of them, while TCM3 gave no amplification for these samples. For the five remaining markers, the amplification efficiency or their sensitivity differed considerably. The TCM7 marker showed the lowest amplification rate with 81.91% of *T. congolense* positive samples amplified, whereas the highest amplification rate was 94.68% for TCM4. TCM1, TCM2 and TCM6 amplified 86.17%, 85.10% and 90.42% of *T. congolense* positive samples, respectively. Details concerning the size of alleles at each microsatellite locus are reported in Additional file 1.

The five markers considered here have shown a genetic diversity in the population of *T. congolense* forest that circulates in tsetse flies of Fontem. This diversity varies according to microsatellite markers (Table 3). For instance, TCM2 was the most polymorphic marker with six alleles while TCM4 was the least polymorphic with only two alleles. For TCM1, TCM6 and TCM7, five alleles were identified for each of them. Some specific alleles such as 240 of TCM1, 190 of TCM2, 180 of TCM4 and 200 of TCM6 were found only in *T. congolense* forest of tsetse flies caught at Menji while the alleles 160 and 177 of TCM7 as well as 173 of TCM6 were found in parasites circulating in tsetse flies of Folepi and Bechati, respectively (Additional file 2).

**Table 3:** **Number of allele and heterozygosity values at each locus**

Loci	TCM1	TCM2	TCM4	TCM6	TCM7
N	5	6	2	5	5
Ho	0.849	0.949	0.011	0.780	0.916
Hs	0.600	0.586	0.011	0.515	0.522

N: Number of alleles at each locus; Ho: observed heterozygosity; Hs: expected heterozygosity.

All alleles identified in *T. congolense* of tsetse flies caught in 2006 were also found in parasites from tsetse flies sampled in 2007 and/or 2009. However, some alleles such as 240 of TCM1, 190 of TCM2, 180 of TCM4, 200 of TCM6 and 177 of TCM7 were identified only in *T. congolense* forest from flies sampled in 2009, while the alleles 173 of TCM6 and 208 of TCM7 were identified only in parasites of tsetse flies caught in 2007 (Additional file 3).

Our results show the highest value of the allelic richness of 2.896 for *T. congolense* populations identified in tsetse flies caught in 2009. In 2006 and 2007, the values of the allelic richness were 2.181 and 2.581, respectively. Comparing the allelic richness according to the years of capture, no significant difference (*P = 0.0715*) was observed. According to villages, the highest value of the allelic richness (*R*_*S*_) of 3.196 was observed for the population of *T. congolense* found in tsetse flies caught at Menji while the parasites identified in tsetse flies caught at Folepi and Bechati recorded *R*_*S*_ values of 2.85 and 2.388, respectively. No significant difference (*P = 0.0655*) was also found for the allelic richness between villages.

### Population genetics analysis

Only single infections of *T. congolense* forest were taken into consideration for these analyses. We considered single infections as samples for which only one allele (homozygote) or two different alleles (heterozygote) were identified for all the five microsatellite markers used. In this context, a sample was considered as having multiple infections if it had more than two alleles for a given locus. TCM2 and TCM7 each revealed one mixed infection while four mixed infections were revealed by TCM1. In addition to multiple infections, all samples showing no amplification for at least three microsatellite loci were excluded for the population genetics analyses. In sum, 87 *T. congolense* forest samples with 78 from Fontem and 9 from DRC were used for the population genetics studies.

**Figure 2:**
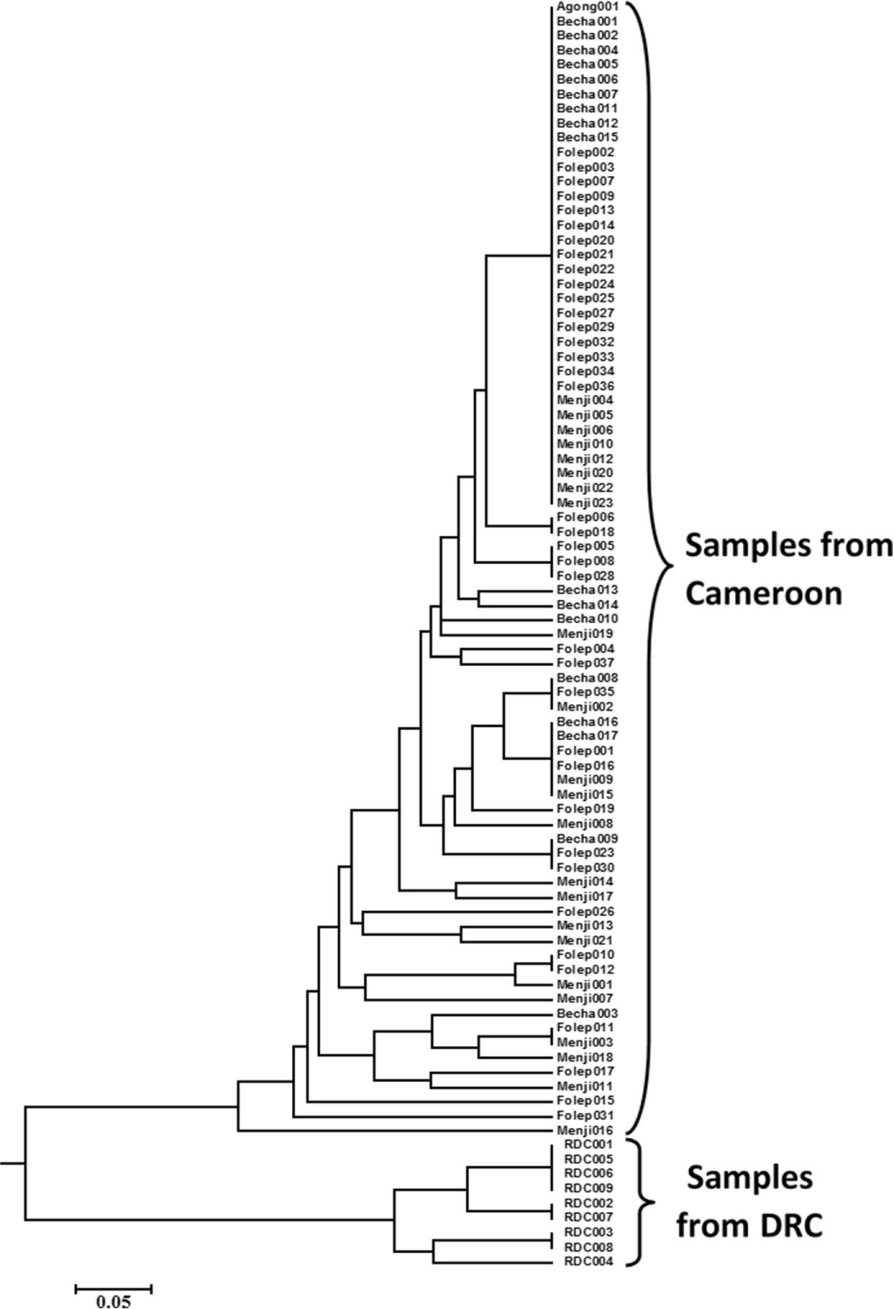
**Dendrogram showing the genetic similarity between** ***T. congolense*** **forest circulating in tsetse flies of different villages of Fontem.**

Multilocus genotypes, obtained for each *T. congolense* forest sample and for the five microsatellites markers considered here, enabled us to determine the level of genetic differentiation between the *T. congolense* forest populations. The genetic analyses performed on the 87 single infections of *T. congolense* forest type revealed low genetic differentiation between samples from different villages or different years of capture. To appreciate the genetic variability within the population of the forest type of *T. congolense* that circulates in tsetse flies of Fontem villages, the multilocus genotypes were used to construct a dendrogram of similarity by calculating Cavalli-Sforza and Edwards [29] chord distances for all pairwise comparisons (Figure 2). Thirty-four different genotypes were obtained for the 87 samples considered for these analyses, indicating inconsistent data with mating reproduction of particular genotypes. About 30 different genotypes were identified in villages of Fontem. However, the genetic distances between samples of Fontem were very low (Figure 2). The dendrogram obtained here can be subdivided into two clusters; the first cluster contains all samples of Fontem villages while the second cluster contains samples of the DRC. The population of *T. congolense* forest that circulates at Fontem seems to form a large single group with little genetic differentiation (Figure 2).

**Figure 3:**
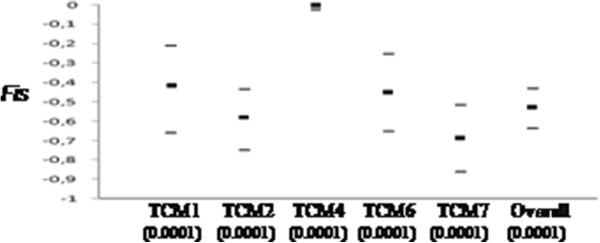
**Individual fixation index (** ***FIS*** **) at Fontem for each locus and overall.** For each locus, the 95% confidence intervals were obtained by Jackknife over populations except for overall loci where it was obtained by bootstraps.

When the samples from different villages are considered as a single population, all the five loci exhibited heterozygote excess as shown by the global in breeding coefficient (*F*_IS_) of -0.52818 (P < 0.0001), which showed a predominant clonal reproduction of *T. congolense* forest (Figure 3). The pairwise *F*_ST_ values between subsamples indicate no genetic differentiation between samples from different villages of Fontem as well as samples from different years of capture (Table 4). However, the *F*_ST_ values obtained between subsamples of Fontem villages and those of the DRC show a greater genetic differentiation (*F*_ST_ ranged from 0.3911 to 0.4212), and thus suggesting a very low migration rate of *T. congolense* between Fontem and DRC.

**Table 4:** ***F***_**ST**_ **value between villages and the Democratic Republic of Congo**

Villages	Folepi	Menji	DRC
Bechati	-0.0068*	-0.0009*	0.4212
Folepi		0.0003*	0.4190
Menji			0.3911

DRC: Democratic Republic of Congo; *P value not significant.

## Discussion

The samples analyzed in this study allowed us to improve our understanding of the population genetics of the forest type of *Trypanosoma congolense*. Both microscopic examination and PCR detected trypanosomes in the mid-guts of tsetse flies of different villages of Fontem in the Southwest Region of Cameroon as already reported by previous authors [18, 32]. The identification of *T. congolense* forest and savannah confirms the results obtained by previous authors who identified these parasites in tsetse flies [32] and domestic animals [15, 33, 34] of different villages of Fontem.

Our results show that *T. congolense* forest has a higher infectious rate than *T. congolense* savannah. The low infection of *T. congolense* savannah is probably due to the level of pathogenicity of this trypanosome subspecies. Indeed, Bengaly *et al.*[35] have shown that Zebu cattle infected by *T. congolense* savannah presented a severe syndrome, which led inexorably to death within four to seven weeks post-infection if no treatment was administered. This probably occurs in the Fonten area since the inhabitants of this area do not use trypanocidal drugs to prevent AAT. This would mean that the number of animals infected by *T. congolense* savannah and on which tsetse flies can become infected by this trypanosome subspecies would be considerably reduced. Moreover, the lack of trypanocidal drug to prevent AAT could explain the increasing trypanosome prevalence from one year to the next (*T. congolense* infection rates have tripled from 2007 to 2009 and quadrupled from 2006 to 2009). These results indicate that for large scale breeding of animals in this area, control measures for AAT at Fontem need to be instituted.

Out of the seven microsatellite markers used in this study, only five gave good amplification for *T. congolense* positive samples from Fontem villages. These results are in line with those of Simo *et al.*[15] who reported identical results when they analyzed samples from domestic animals of the same villages. The fact that only few samples were amplified by TCM5 is also in line with observations of Simo *et al.*[15] who reported similar results in domestic animals of the same area. However, the amplification of nine samples from the DRC by this marker indicates that some genetic differences are probably due to mutations at the primer binding sites in trypanosomes circulating in villages of Fontem. This would mean that TCM5 seems to be an unsuitable genetic marker for subsequent investigations on the genetic characterization of the forest type of *T. congolense*. TCM3 neither amplified the Fontem samples nor those from the DRC. These results corroborate those obtained by Morrison *et al.*[14] and Simo *et al.*[15] and suggest TCM3 as a suitable marker for the differentiation of savannah and forest types of *T. congolense*. The variations in the size of alleles for each microsatellite locus corroborate results obtained by Morrison *et al.*[14] and Simo *et al.*[15] on *T. congolense* samples of Gambia and Cameroon respectively. The great disparity between the sizes of alleles observed here and for almost all the microsatellite data suggests that the microsatellite evolution within trypanosomes does not occur in a strictly stepwise manner.

Most alleles identified in this study were found in all villages. These alleles belong to major genotypes and indicate the circulation of these major genotypes in all villages of Fontem. Beside these major genotypes, minor genotypes were also observed mainly in trypanosomes found in tsetse flies caught at Menji. These results are in line with those obtained in domestic animals of the same area [15]. For instance, the allele 180 bp of TCM4 was identified in tsetse flies of Menji only. Recently, the same allele was identified in pigs of the same village, confirming the circulation of the same minor and major genotypes between animals and tsetse flies.

Both mixed and multiple (infection of different types of different species or subspecies of trypanosomes) infections were found in our study, corroborating the results of Morlais *et al.*[32] and Malele *et al.*[36] who have shown that infected tsetse flies from Cameroon and other zones of Africa frequently harbor more than one trypanosome species or subspecies. The distribution of alleles according to the year of capture shows that the number of new alleles has increased from 17 in 2007 to 21 in 2009. This increasing number of alleles could be explained by genetic exchanges, which may occur between different strains or mutations at some microsatellite loci. Indeed, many genotypes of the same species in the midguts of tsetse flies seem to be a principal cause of the appearance of new genotypes within species having mainly a clonal reproduction [37].

The population genetic analyses revealed low genetic variability between isolates of *T. congolense* forest that circulate in tsetse flies caught in the same village and in different villages of Fontem. This result is in line with results reported by Simo *et al.*[15] in domestic animals of the same locality. The heterozygote excess (Table 4), as revealed by the *F*_IS_ values (Figure 3), permit us to conclude in favor of a clonal reproduction of *T. congolense*. These results corroborate those of Tibayrenc *et al.*[13] and Simo *et al.*[15]. However, using the same microsatellite markers, Morrison *et al.*[14] demonstrated that mating apparently occurs in *T. congolense*, although it is not obligatory. There is, therefore, some controversy on the population genetics of *T. congolense* that requires further investigation in different areas of Africa in order to have a clearer understanding of the genetic structure of this parasite.

The analysis of the dendrogram in Figure 2 showed that the *T. congolense* sub-population of the DRC is genetically different from that of Cameroon. Indeed, the strains of the sub-population of DRC form one cluster while all strains of the sub-population of Fontem are grouped in another big cluster with low genetic distances between strains of DRC or strains of Fontem villages. However, important genetic distances were observed between the strains of DRC and those of Fontem, as illustrated by the dendrogram showing the genetic similarities between strains (Figure 2). The genetic differentiation between the samples of the DRC and those of Cameroon was strongly significant as shown by the *F*_ST_ value of 0.39 (P < 0.0001). These results show a perfect sub-structuring between samples of the DRC and those of Cameroon. This important sub-structuration could be explained by the geographic distance between these two countries or between the sampling areas. There was no sub-structuration between different subpopulations of the forest type of *T. congolense* that circulates in tsetse flies of different villages of Fontem as shown by the low and non-significant difference between the *F*_ST_ values of different villages and different years of capture. This is highlighted by the heterogeneity in the distribution of samples in different nodes or clusters of the dendrogram (Figure 2) that illustrates genetic similarities between strains. The absence of sub-structuration within the populations of *T. congolense* that infect tsetse flies of different villages of Fontem suggests that the same genotypes or genotype families circulate in this area.

## Conclusion

The results of this study have shown that the infection rates of *T. congolense* vary significantly between the populations of tsetse flies of different villages of Fontem as well as the years of capture. The microsatellite markers showed a low genetic variability within Fontem samples. Our results on the population genetics of *T. congolense* forest plead in favor of a predominant clonal reproduction of these parasites. No sub-structuring was observed between subpopulations of different villages of Fontem although with DRC samples, a perfect structuration was observed, certainly due to geographical distance between the two sampling areas.

## Electronic supplementary material

Additional file 1: **Characteristics of** ***Trypanosoma congolense*** **forest positive samples, size of alleles and the minimum number of genotypes at each microsatellite locus.** (DOC 224 KB)

Additional file 2: **Allelic frequency for each locus and for each village.** (DOC 91 KB)

Additional file 3: **Allelic frequency for each locus and for each year of capture.** (DOC 92 KB)
