# Supplementary material for: Population genetics of forest type of Trypanosoma congolense circulating in Glossina palpalis palpalis of Fontem in the South-West region of Cameroon
Source: Parasit Vectors. 2014 Aug 20;7:385. doi: 10.1186/1756-3305-7-385 (PMC4261900; doi:10.1186/1756-3305-7-385)
Supplement: Supplementary file 1 — Additional file 1: Characteristics of Trypanosoma congolense forest positive samples, size of alleles and the minimum number of genotypes at each microsatellite locus. (DOC 224 KB) [file 13071_2014_1642_MOESM1_ESM.doc]

Additional file 1: Characteristics of *Trypanosoma congolense* forest positive samples, size of alleles and the minimum number of genotypes at each microsatellite locus.

| **Samples** | **Villages** | **Years** | **Species** | **Sex** | **Para** | **TCM1** | **TCM2** | **TCM4** | **TCM6** | **TCM7** | **MNG** |
| --- | --- | --- | --- | --- | --- | --- | --- | --- | --- | --- | --- |
| 490 | Agong | 2006 | G. pal | F | - | 188/215 | 183/205 | 152/152 | 180/214 | 162/186 | 1 |
| 177 | Bechati | 2006 | G. pal | M | - | 188/215 | 183/205 | 152/152 | 180/214 | 162/186 | 1 |
| 145 | Bechati | 2006 | G. pal | F | - | 188/215 | 183/205 | 152/152 | 180/214 | 162/186 | 1 |
| 124 | Bechati | 2006 | G. pal | F | - | 194/194 | 205/240 | 152/152 | 180/214 | 162/186 | 1 |
| 120 | Bechati | 2006 | G. pal | F | - | 188/215 | 183/205 | 152/152 | 180/214 | 162/186 | 1 |
| 123 | Bechati | 2006 | G. pal | F | - | 188/215 | 183/205 | 152/152 | 180/214 | 162/186 | 1 |
| 2315 | Bechati | 2007 | G. pal | M | - | 188/215 | 183/205 | 152/152 | 173/214 | 162/186 | 1 |
| 2264 | Bechati | 2007 | G. pal | F | + | 188/215 | 183/205 | 152/152 | 180/214 | 162/186 | 1 |
| 2310 | Bechati | 2007 | G. pal | M | + | 188/215 | 183/205 | 152/152 | 180/180 | 162/162 | 1 |
| 2279 | Bechati | 2007 | G. pal | M | - | 188/215 | 183/205 | 152/152 | 180/214 | 162/186 | 1 |
| 2306 | Bechati | 2007 | G. pal | F | + | 188/188 | 183/205 | 152/152 | 180/180 | 162/186 | 1 |
| 2202 | Bechati | 2007 | G. pal | F | - | 188/215 | 183/205 | 152/152 | 173/173 | 162/186/208 | 2 |
| 2262 | Bechati | 2007 | G. pal | M | - | 000/000 | 183/205 | 152/152 | 180/214 | 162/186 | 1 |
| 71-29 | Bechati | 2009 | G. pal | M | - | 188/215 | 183/183 | 152/152 | 180/214 | 162/186 | 1 |
| 71-39 | Bechati | 2009 | G. pal | M | + | 188/215 | 183/240 | 152/152 | 180/214 | 162/186 | 1 |
| 71-13 | Bechati | 2009 | G. pal | F | - | 188/215 | 183/205 | 152/152 | 180/214 | 162/186 | 1 |
| 71-42 | Bechati | 2009 | G. pal | F | - | 188/215 | 183/205 | 152/152 | 180/214 | 162/186 | 1 |
| 73-21 | Bechati | 2009 | G. pal | F | - | 188/215 | 183/205 | 152/152 | 180/180 | 162/186 | 1 |
| 72-19 | Bechati | 2009 | G. pal | F | - | 188/215 | 183/205 | 152/152 | 180/180 | 162/186 | 1 |
| 72-2 | Bechati | 2009 | G. pal | M | - | 000/000 | 000/000 | 000/000 | 185/214 | 000/000 | 1 |
| 443 | Folepi | 2006 | G. pal | M | - | 188/188 | 183/205 | 152/152 | 180/214 | 162/186 | 1 |
| 218 | Folepi | 2006 | G. pal | M | - | 188/215 | 183/205 | 152/152 | 180/180 | 162/186 | 1 |
| 316 | Folepi | 2006 | G. pal | M | - | 166/188/215 | 183/205 | 152/152 | 180/214 | 162/186 | 2 |
| 257 | Folepi | 2006 | G. pal | F | - | 188/215 | 183/205 | 152/152 | 180/214 | 162/186 | 1 |
| 295 | Folepi | 2006 | G. pal | F | - | 188/215 | 183/205 | 152/152 | 180/214 | 162/186 | 1 |
| 323 | Folepi | 2006 | G. pal | F | - | 188/215 | 205/205 | 152/152 | 180/214 | 000/000 | 1 |
| 1116 | Folepi | 2007 | G. pal | M | - | 188/215 | 183/205 | 152/152 | 180/214 | 162/186 | 1 |
| 1653 | Folepi | 2007 | G. pal | F | - | 188/215 | 183/205 | 152/152 | 180/214 | 162/186 | 1 |
| 1835 | Folepi | 2007 | G. pal | F | - | 188/215 | 183/205 | 152/152 | 180/214 | 162/186 | 1 |
| 1537 | Folepi | 2007 | G. pal | M | - | 188/215 | 183/205 | 152/152 | 180/180 | 162/186 | 1 |
| 1129 | Folepi | 2007 | G. pal | M | - | 188/215 | 183/205 | 152/152 | 180/214 | 162/186 | 1 |
| 1450 | Folepi | 2007 | G. pal | M | - | 188/215 | 183/205 | 152/152 | 180/214 | 162/186 | 1 |
| 1506 | Folepi | 2007 | G. pal | F | - | 188/215 | 183/183 | 152/152 | 185/185 | 162/186 | 1 |
| 1631 | Folepi | 2007 | G. pal | M | - | 166/188 | 183/205 | 152/152 | 180/180 | 162/186 | 1 |
| 1657 | Folepi | 2007 | G. pal | M | - | 188/215 | 183/205 | 152/152 | 180/214 | 162/186 | 1 |
| 2037 | Folepi | 2007 | G. pal | F | - | 188/215 | 183/205 | 152/152 | 180/180 | 162/162 | 1 |
| 1119 | Folepi | 2007 | G. pal | M | - | 188/188 | 183/205 | 152/152 | 180/214 | 162/186 | 1 |
| 1905 | Folepi | 2007 | G. pal | F | + | 188/188 | 183/183 | 152/152 | 180/180 | 162/186 | 1 |
| 1680 | Folepi | 2007 | G. pal | F | - | 188/215 | 183/205 | 152/152 | 180/180 | 162/162 | 1 |
| 1002 | Folepi | 2007 | G. pal | F | - | 188/215 | 183/205 | 152/152 | 180/214 | 162/162 | 1 |
| 1970 | Folepi | 2007 | G. pal | F | - | 188/215 | 183/205 | 152/152 | 180/214 | 162/186 | 1 |
| 1406 | Folepi | 2007 | G. pal | F | - | 188/215 | 194/217 | 152/152 | 180/214 | 186/186 | 1 |
| 2031 | Folepi | 2007 | G. pal | F | - | 188/215 | 183/205 | 152/152 | 180/214 | 162/186 | 1 |
| 1340 | Folepi | 2007 | G. pal | F | - | 194/194 | 183/205 | 152/152 | 180/214 | 162/186 | 1 |
| 1075 | Folepi | 2007 | G. pal | M | - | 166/194/215 | 183/205 | 152/152 | 180/214 | 162/186 | 2 |
| 2049 | Folepi | 2007 | G. pal | F | - | 188/215 | 183/205 | 152/152 | 180/214 | 162/186 | 1 |
| 1823 | Folepi | 2007 | G. pal | M | - | 188/215 | 183/205 | 152/152 | 180/214 | 162/186 | 1 |
| 1602 | Folepi | 2007 | G. pal | F | - | 188/215 | 183/205 | 152/152 | 180/214 | 162/162 | 1 |
| 2029 | Folepi | 2007 | G. pal | F | - | 188/188 | 183/205 | 152/152 | 180/214 | 162/186 | 1 |
| 1677 | Folepi | 2007 | G. pal | F | - | 188/215 | 183/205 | 152/152 | 180/214 | 162/186 | 1 |
| 1587 | Folepi | 2007 | G. pal | F | - | 194/194 | 183/183 | 152/152 | 000/000 | 000/000 | 1 |
| 2042 | Folepi | 2007 | G. pal | F | - | 188/215 | 000/000 | 152/152 | 180/180 | 169/208 | 1 |
| 1228 | Folepi | 2007 | G. pal | F | - | 000/000 | 194/217 | 152/152 | 180/214 | 000/000 | 1 |
| 1415 | Folepi | 2007 | G. pal | F | - | 188/215 | 000/000 | 152/152 | 180/214 | 000/000 | 1 |
| 82-15 | Folepi | 2009 | G. pal | F | - | 188/215 | 183/205 | 152/152 | 180/214 | 162/186 | 1 |
| 83-12 | Folepi | 2009 | G. pal | F | - | 188/215 | 183/205 | 152/152 | 180/214 | 162/186 | 1 |
| 83-8 | Folepi | 2009 | G. pal | M | - | 188/215 | 183/205 | 152/152 | 180/214 | 169/177 | 1 |
| 83-52 | Folepi | 2009 | G. pal | F | + | 188/215 | 000/000 | 152/152 | 180/214 | 162/186 | 1 |
| 83-13 | Folepi | 2009 | G. pal | F | - | 000/000 | 000/000 | 152/152 | 180/180 | 162/186 | 1 |
| 83-14 | Folepi | 2009 | G. pal | F | - | 000/000 | 000/000 | 152/152 | 000/000 | 000/000 | 1 |
| 84-21 | Folepi | 2009 | G. pal | M | - | 000/000 | 000/000 | 152/152 | 000/000 | 000/000 | 1 |
| 83-19 | Folepi | 2009 | G. pal | F | - | 194/215 | 000/000 | 152/152 | 000/000 | 000/000 | 1 |
| 85-11 | Folepi | 2009 | G. pal | M | - | 000/000 | 000/000 | 152/152 | 000/000 | 000/000 | 1 |
| 83-40 | Folepi | 2009 | G. pal | F | - | 188/215 | 000/000 | 000/000 | 000/000 | 000/000 | 1 |
| 82-16 | Folepi | 2009 | G. pal | F | - | 000/000 | 000/000 | 000/000 | 185/185 | 000/000 | 1 |
| 2446 | Menji | 2007 | G. pal | F | - | 188/188 | 183/205 | 152/152 | 180/180 | 162/186 | 1 |
| 2471 | Menji | 2007 | G. pal | F | - | 188/215 | 194/217 | 152/152 | 180/214 | 162/186 | 1 |
| 2542 | Menji | 2007 | G. pal | M | - | 194/194 | 183/205 | 152/152 | 180/214 | 162/186 | 1 |
| 2537 | Menji | 2007 | G. pal | M | - | 188/215 | 183/205 | 152/152 | 000/000 | 000/000 | 1 |
| 2549 | Menji | 2007 | G. pal | F | + | 000/000 | 183/205 | 152/152 | 180/214 | 000/000 | 1 |
| 2531 | Menji | 2007 | G. pal | F | - | 188/215 | 183/205 | 152/152 | 180/214 | 162/186 | 1 |
| 2583 | Menji | 2007 | G. pal | F | - | 000/000 | 000/000 | 000/000 | 000/000 | 000/000 |  |
| 53-38 | Menji | 2009 | G. pal | F | - | 166/188/240 | 183/205 | 152/152 | 180/180 | 162/186 | 2 |
| 53-47 | Menji | 2009 | G. pal | F | - | 166/215/240 | 183/183 | 152/152 | 180/214 | 162/186 | 2 |
| 53-46 | Menji | 2009 | G. pal | M | - | 188/215 | 183/205 | 152/152 | 180/180 | 162/186 | 1 |
| 53-24 | Menji | 2009 | G. pal | F | - | 194/194 | 183/205 | 152/152 | 185/185 | 162/186 | 1 |
| 61-3 | Menji | 2009 | G. pal | M | - | 188/215 | 183/205 | 152/152 | 180/214 | 162/186 | 1 |
| 63-31 | Menji | 2009 | G. pal | M | - | 188/215 | 183/205 | 152/152 | 180/214 | 162/186 | 1 |
| 60-15 | Menji | 2009 | G. pal | F | - | 194/215 | 183/205 | 152/152 | 180/214 | 162/186 | 1 |
| 53-13 | Menji | 2009 | G. pal | M | - | 194/194 | 183/205 | 152/152 | 180/180 | 162/186 | 1 |
| 53-44 | Menji | 2009 | G. pal | F | - | 188/215 | 190/205 | 152/152 | 180/214 | 162/186 | 1 |
| 58-14 | Menji | 2009 | G. pal | F | - | 188/215 | 190/217 | 152/152 | 180/214 | 162/186 | 1 |
| 53- | Menji | 2009 | G. pal |  |  | 188/215 | 183/217 | 152/152 | 180/214 | 162/162 | 1 |
| 53-15 | Menji | 2009 | G. pal | M | - | 188/215 | 183/205 | 152/152 | 180/180 | 162/186 | 1 |
| 53-37 | Menji | 2009 | G. pal | F | - | 188/188 | 194/205 | 152/152 | 180/214 | 162/186 | 1 |
| 53-18 | Menji | 2009 | G. pal | F | - | 188/215 | 183/205 | 152/152 | 180/214 | 162/186 | 1 |
| 53-52 | Menji | 2009 | G. pal | F | - | 188/215 | 183/205/240 | 152/152 | 180/214 | 162/186 | 2 |
| 53-30 | Menji | 2009 | G. pal | M | - | 188/215 | 183/205 | 152/152 | 180/214 | 162/186 | 1 |
| 53-8 | Menji | 2009 | G. pal | M | + | 166/194 | 183/205 | 152/180 | 180/214 | 162/162 | 1 |
| 60-16 | Menji | 2009 | G. pal | F | - | 188/215 | 183/205 | 152/152 | 180/200 | 162/186 | 1 |
| 63-23 | Menji | 2009 | G. pal | F | - | 000/000 | 194/205 | 152/152 | 180/180 | 162/186 | 1 |
| 60-17 | Menji | 2009 | G. pal | F | - | 000/000 | 000/000 | 152/152 | 000/000 | 000/000 | 1 |
| 63-24 | Menji | 2009 | G. pal | F | - | 188/215 | 183/205 | 152/152 | 180/214 | 000/000 | 1 |
| 58-27 | Menji | 2009 | G. pal | M | - | 000/000 | 000/000 | 000/000 | 180/214 | 000/000 | 1 |
| J163K10 | DRC | 2011 | G. f q | / | / | 166/200 | 183/225 | 156/175 | 185/214 | 162/186 | 1 |
| 220 | DRC | 2011 | G. f. q | / | / | 166/200 | 183/225 | 156/175 | 185/214 | 162/186 | 1 |
| A200 | DRC | 2011 | G. f. q | / | / | 166/200 | 183/225 | 156/175 | 185/214 | 162/186 | 1 |
| J170 | DRC | 2011 | G. f. q | / | / | 166/200 | 183/183 | 156/175 | 185/214 | 000/000 | 1 |
| J175 | DRC | 2011 | G. f. q | / | / | 166/200 | 183/225 | 156/175 | 185/214 | 000/000 | 1 |
| 229 | DRC | 2011 | G. f. q | / | / | 166/200 | 183/205 | 156/175 | 185/214 | 162/190 | 1 |
| J165 | DRC | 2011 | G. f. q | / | / | 166/166 | 183/225 | 156/175 | 185/214 | 162/186 | 1 |
| 222 | DRC | 2011 | G. f. q | / | / | 166/166 | 183/225 | 156/175 | 185/214 | 000/000 | 1 |
| 227 | DRC | 2011 | G. f. q | / | / | 166/200 | 183/183 | 156/175 | 185/214 | 162/162 | 1 |

Para: parasitological examination; G. pal: *Glossina palpalis palpalis*; G. f. q: *Glossina fuscipes quanzensis*; F: female; M: male. MNG: Minimun Number of Genotypes; DRC: Democratic Republic of Congo.
