# Supplementary figures and images for: Population genetics of forest type of Trypanosoma congolense circulating in Glossina palpalis palpalis of Fontem in the South-West region of Cameroon
Source: Parasit Vectors. 2014 Aug 20;7:385. doi: 10.1186/1756-3305-7-385 (PMC4261900; doi:10.1186/1756-3305-7-385)

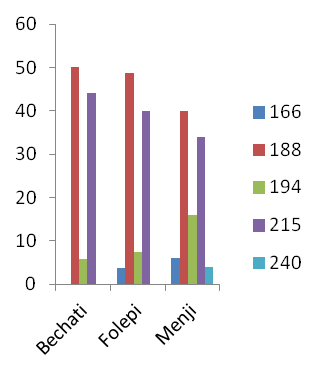

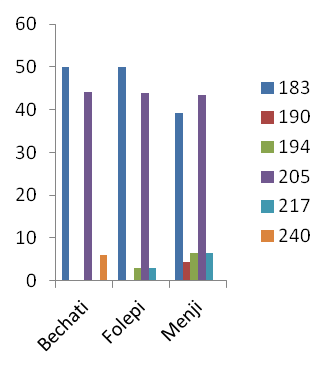

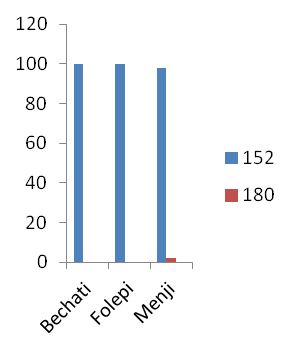

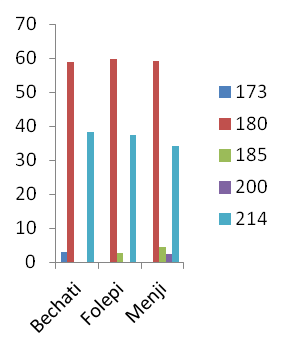

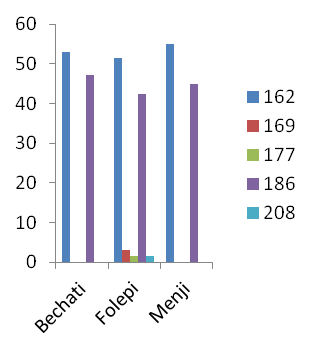


TCM2

TCM1

TCM6

TCM7

TCM4

**Additional file 2: Allelic frequency for each locus and for each village**

Supplement: Supplementary file 2 — Additional file 2: Allelic frequency for each locus and for each village. (DOC 91 KB) [file 13071_2014_1642_MOESM2_ESM.doc]
