# Supplementary material for: Population genetics of forest type of Trypanosoma congolense circulating in Glossina palpalis palpalis of Fontem in the South-West region of Cameroon
Source: Parasit Vectors. 2014 Aug 20;7:385. doi: 10.1186/1756-3305-7-385 (PMC4261900; doi:10.1186/1756-3305-7-385)
Supplement: Supplementary file 3 — Additional file 3: Allelic frequency for each locus and for each year of capture. (DOC 92 KB) [file 13071_2014_1642_MOESM3_ESM.doc]

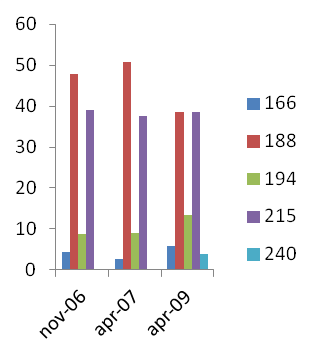

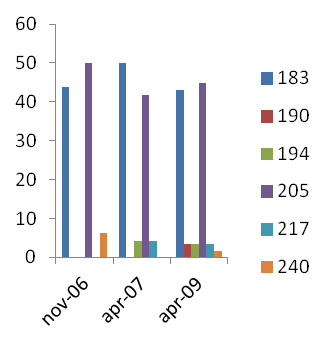

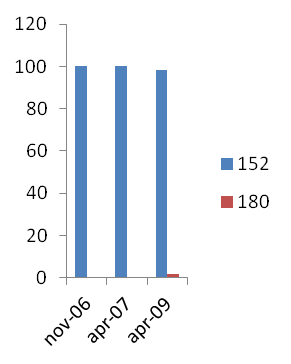

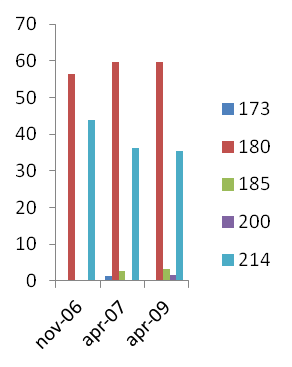

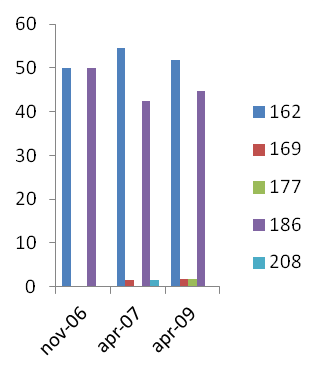


TCM6

TCM2

TCM1

TCM4

TCM7

Additional file 3: Allelic frequency for each locus and for each year of capture
